# Novel thoracoscopic approach to posterior mediastinal goiters: report of two cases

**DOI:** 10.1186/1749-8090-3-55

**Published:** 2008-10-07

**Authors:** Faisal Al-Mufarrej, Marc Margolis, Barbara Tempesta, Eric Strother, Farid Gharagozloo

**Affiliations:** 1 The George Washington University Medical Center, Department of Surgery, 2300 Eye Street N.W., Washington, D.C. 20037, USA; 2 Washington Institute of Thoracic and Cardiovascular Surgery, 2175 K Street N.W., Washington D.C 20037, USA

## Abstract

Trans-cervical resection of posterior mediastinal goiters is usually very difficult, requiring a high thoracotomy. Until recently, using conventional video-assisted thoracoscopic surgery to resect such tumors has been technically difficult and unsafe. By virtue of 3 dimensional visualization, greater dexterity, and more accurate dissection, the Da Vinci robot, for the first time, enables a completely minimally invasive approach to the posterior superior mediastinum.

## Background

Mediastinal goiters are goiters that extent beyond the thoracic outlet into the mediastinum. Most mediastinal goiters are retrosternally situated in the anterior mediastinal compartment. Posterior mediastinal goiters, either retrotracheal or retroesophageal, are rare, making up 4% of all mediastinal goiters [1]. Surgery for mediastinal goiters should always be considered, even in elderly patients because of the high risk of tracheal compression and the low morbidity of the surgery.

Most anterior mediastinal goiters are benign and can be removed through a cervical approach. Few anterior mediastinal goiters require a sternotomy as well for complete resection; those tumors are usually very large, aberrant, malignant, or found in patients undergoing re-operative thyroid surgery [2]. On the other hand, trans-cervical resection of posterior mediastinal goiters is usually very difficult, requiring a high thoracotomy. Using conventional video-assisted thoracoscopic surgery (VATS) to resect such tumors is technically difficult and unsafe. Despite that, there has been a continued interest in limiting the extent of surgery on patients with posterior mediastinal goiters by combining thoracoscopic surgery with a limited open cervico-thoracic approach [3]. By virtue of 3 dimensional visualization, greater dexterity, and more accurate dissection, the Da Vinci robot, for the first time, enables a completely minimally invasive approach to the posterior superior mediastinum.

## Case presentation

### Case 1

**Figure 1:**
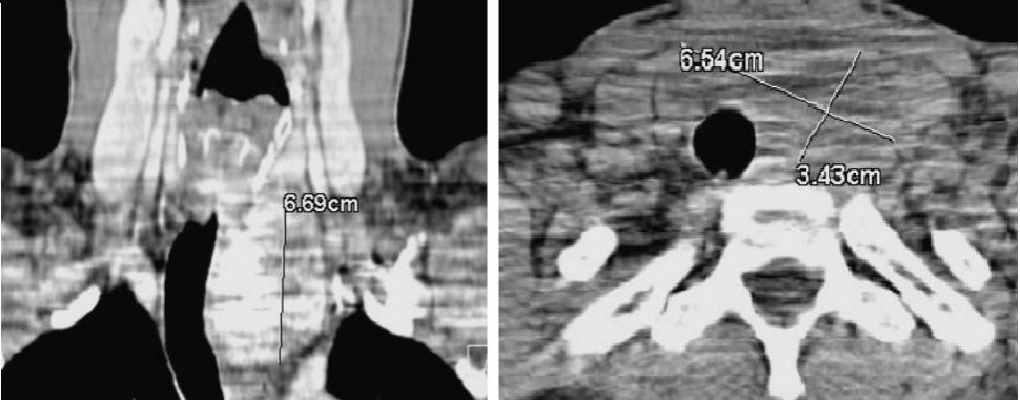
CT of the neck and chest confirming a posterior mediastinal mass arising from the left thyroid lobe at level of the thoracic outlet.

A 35 year-old gentleman with past medical history of hypertension presented with complaints of intermittent shortness of breath. On palpation, the thyroid was normal. Computed axial tomography (CT) of the neck and chest confirmed a 6.7 × 6.5 × 3.4 cm posterior mediastinal mass extending from the thoracic outlet, probably the left thyroid lobe, all the way down to the aortic arch with tracheal compression and deviation (Figure 1). The patient was advised to undergo a combined procedure starting with a left-sided robot-assisted thoracoscopic dissection of the mass followed by trans-cervical enucleation and possible thyroidectomy.

Following general anesthesia and successful double-lumen endotracheal intubation, the patient was positioned in a right lateral position with his left side up. Three 2-cm incisions were made. Dissection was carried into the left pleural space. The left lung was deflated, and the patient was maintained on one-lung ventilation. The camera was introduced into the pleural cavity through the middle incision, and the robot's left and right arms were positioned from the anterior and posterior incisions. The lung was retracted, opening the sub-carinal space, and exposure was maintained with an endoscopic self-retaining retractor placed through a fourth small mid-axillary incision in the seventh intercostal space. With the aid of the Da Vinci robot, the mediastinal mass was then dissected anteriorly off the aortic arch, the left subclavian artery, and the left carotid artery. The blood supply of the intra-thoracic thyroid originated from the internal mammary artery which was carefully dissected off the mass with cautery of its feeding branches. The mass was then freed caudally and posteriorly where the recurrent laryngeal nerve was identified. After ensuring hemostasis, a posterior chest tube was left in place and the incisions were closed.

The patient was then positioned in a supine position and successfully re-intubated with a single lumen nerve integrity monitor endotracheal tube for continuous electromyography of the larynx. Upon cervical exploration, the mass was confirmed to be arising from the left lower pole of the thyroid. Dissection was carried down into the mediastinum, and the mass mobilized at its lowest edge. The mass was then retracted superiorly and carefully dissected from lateral to medial. After delivering the mass into our transverse cervical incision, a left thyroid lobectomy was performed.

Pathological assessment of the mass confirmed multinodular goiter.

Patient did well post-operatively and was discharged once his chest tube output was low enough to be discontinued.

### Case 2

**Figure 2:**
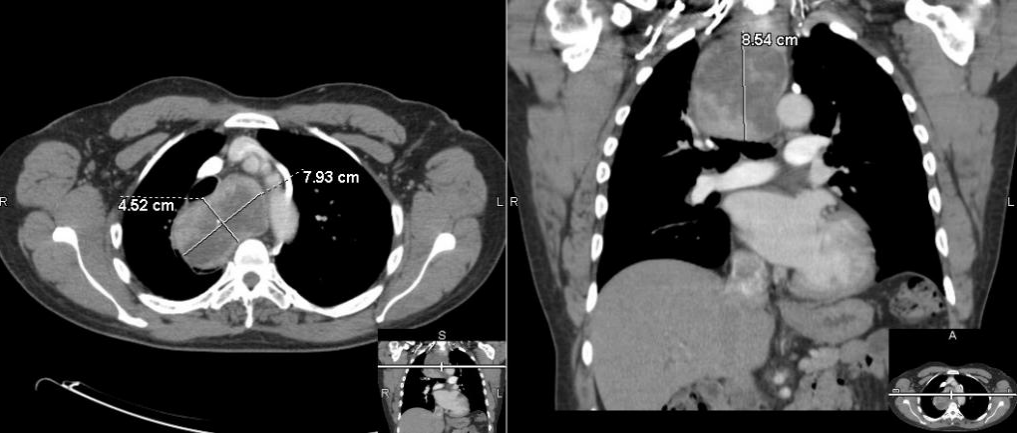
CT of the chest revealing a posterior mediastinal mass that extends all the way down to the aortic arch with tracheal compression and deviation.

A 74 year old gentleman with past medical history of right hemi-thyroidectomy for a benign right thyroid nodule was noted to have significant tracheal deviation on a chest roentenogram done for chest discomfort during exercise. A left lower pole thyroid mass was barely palpable on examination of the neck. A CT of the chest revealed an 8.5 × 7.9 × 4.5 cm posterior mediastinal mass arising from the left lower pole of the thyroid and extending down into the right posterior mediastinum at the level of the aortic arch causing tracheal deviation to the left with 70% compression (Figure 2). The patient was advised to undergo thoraco-cervical resection of the mediastinal goiter and completion thyroidectomy.

**Figure 3:**
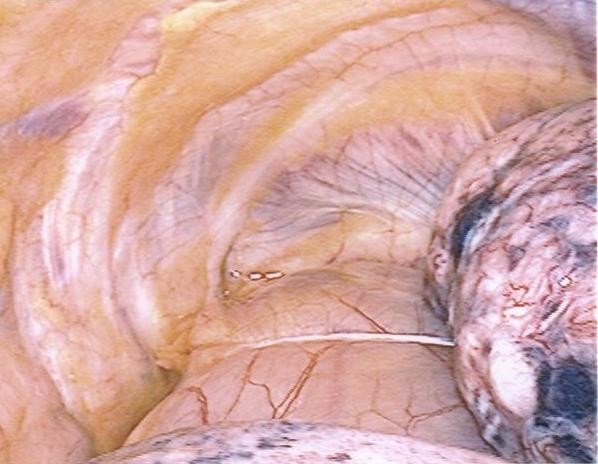
Inferior portion of the goiter extending into the posterior mediastinum as visualized on thoracoscopy.

The patient underwent a procedure similar to that described in Case 1; however, a right sided thoracoscopic approach was taken given the location of the goiter in the mediastinum. The goiter (Figure 3) was also too large to be delivered trans-cervically, so after complete thoraco-cervical dissection and mobilization, the mass was delivered trans-cervically down into the posterior mediastinum. The cervical incision was closed. The patient was re-intubated and re-positioned. The thoracic incisions were reopened, and the mass was delivered through one of them using conventional thoracoscopy.

Pathological assessment of the mediastinal mass confirmed multinodular goiter with no parathyroid tissue in the specimen; however, the patient was hypocalcemic post-operatively and remains so to date. And while his voice was strong at discharge, the patient noted vocal weakness and fatiguability on follow-up and eventually underwent left thyroplasty for persistent left vocal cord paresis.

## Conclusion

The desire to find a minimally invasive approach to posterior mediastinal tumors stems from increasing evidence that thoracoscopic surgery is associated with quicker recovery and less morbidity than open surgery. Evidence suggests that VATS patients have significantly less early postoperative pain, fewer chest tube days, shorter hospital length of stay, and faster return to work [4,5]. Evidence also suggests that VATs patients have better oxygenation [6], improved pulmonary function [7] and less postoperative pneumonia [5] than conventional thoracotomy patients. A VATS approach results in a significantly lower overall complication and mortality rate. One study reported a 45% complication rate and a 3.6% mortality rate with thoracotomies compared with a 28% complication rate and a 0% mortality rate associated with VATS [8]. Furthermore, after the first postoperative week and within the first post-operative year, despite similar pain medication requirements, VATS patients have less chronic pain and subjective shoulder dysfunction than thoracotomy patients [9]. Finally, VATS patients tend to have more confidence regarding wound size and their overall impression of the operation [10].

A number of reports have been published concerning the surgical treatment of substernal goiters. Endocrine surgeons utilize an extra-cervical approach approximately 11% of the time to remove a substernal goiter, with sternotomies used about two thirds of the time and a thoracotomy used in the remaining cases [1]. As it is with Case 2, about 5% to 37% of operations for sub-sternal goiters are performed because of recurrent or persistent disease after a sub-total or hemi-thyroidectomy [11]. Most described cervico-thoracic cases in the literature start with a cervical dissection followed by a thoracic approach. With a robotic approach, whether the tumor is small enough to be delivered cervically or not, we suggest starting with the thoracic portion of the dissection so that any limitations in the dissection and tumor mobilization may be addressed in the open cervical portion of the procedure.

As demonstrated in Case 2, the risks of post-operative hypoparathyroidism and vocal cord paralysis with substernal goiters are usually higher than with cervical goiters [11]. Interestingly, however, evidence suggests that there is no significant difference in the incidence of these complications between the proportion of patients who do or do not undergo sternotomy or thoracotomy, suggesting that the injury may be occurring more often in the cervical portion of the procedure than the thoracic portion [12]. The reported mean postoperative hospital stay for all patients undergoing substernal goiters resection ranges from 5 to 10 days [12,13]. Our patients' hospital stays were 4 and 7 days long, respectively.

The Da Vinci robotic technology offers a novel minimally invasive approach to substernal goiters extending into the posterior mediastinum. It appears to be safe and, like VATS, may offer an advantage over an open thoracic approach. Further studies will be required to define its precise role in thyroid surgery.

## Consent

Written informed consent was obtained from the patients for publication of this case report and the accompanying image. A copy of the written consent is available for review by the Editor-in-Chief of this journal.

## Competing interests

The authors declare that they have no competing interests.

## Authors' contributions

FA-M, MM, and FG as well as ES, SA, were all involved in the technical aspects of the surgeries described above. FA-M did the background literature search and drafted the manuscript. BT was involved in the patients' peri-operative care and prepared the images for the manuscript. MM and FG were involved in the critical review of the intellectual content of the manuscript. All authors have read and approved the final manuscript.
